# M_5_ Muscarinic Receptors Mediate Striatal Dopamine Activation by Ventral Tegmental Morphine and Pedunculopontine Stimulation in Mice

**DOI:** 10.1371/journal.pone.0027538

**Published:** 2011-11-15

**Authors:** Stephan Steidl, Anthony D. Miller, Charles D. Blaha, John S. Yeomans

**Affiliations:** 1 Department of Psychology, University of Toronto, Toronto, Ontario, Canada; 2 Department of Psychology, University of Memphis, Memphis, Tennessee, United States of America; 3 Centre for Biological Timing and Cognition, University of Toronto, Toronto, Ontario, Canada; University of Granada, Spain

## Abstract

Opiates, like other addictive drugs, elevate forebrain dopamine levels and are thought to do so mainly by inhibiting GABA neurons near the ventral tegmental area (VTA), in turn leading to a disinhibition of dopamine neurons. However, cholinergic inputs from the laterodorsal (LDT) and pedunculopontine (PPT) tegmental nucleus to the VTA and substantia nigra (SN) importantly contribute, as either LDT or PPT lesions strongly attenuate morphine-induced forebrain dopamine elevations. Pharmacological blockade of muscarinic acetylcholine receptors in the VTA or SN has similar effects. M_5_ muscarinic receptors are the only muscarinic receptor subtype associated with VTA and SN dopamine neurons. Here we tested the contribution of M_5_ muscarinic receptors to morphine-induced dopamine elevations by measuring nucleus accumbens dopamine efflux in response to intra-VTA morphine infusion using *in vivo* chronoamperometry. Intra-VTA morphine increased nucleus accumbens dopamine efflux in urethane-anesthetized wildtype mice starting at 10 min after infusion. These increases were absent in M_5_ knockout mice and were similarly blocked by pre-treatment with VTA scopolamine in wildtype mice. Furthermore, in wildtype mice electrical stimulation of the PPT evoked an initial, short-lasting increase in striatal dopamine efflux, followed 5 min later by a second prolonged increase in dopamine efflux. In M_5_ knockout mice, or following systemic pre-treatment with scopolamine in wildtype mice, the prolonged increase in striatal dopamine efflux was absent. The time course of increased accumbal dopamine efflux in wildtype mice following VTA morphine was consistent with both the prolonged M_5_-mediated excitation of striatal dopamine efflux following PPT electrical stimulation and accumbal dopamine efflux following LDT electrical stimulation. Therefore, M_5_ receptors appear critical for prolonged PPT excitation of dopamine efflux and for dopamine efflux induced by intra-VTA morphine.

## Introduction

Dopamine neurotransmission in the forebrain plays an important role in the reinforcing effects of drugs of abuse [1]. Opiate drugs like heroin and morphine are thought to affect dopaminergic neurotransmission through their actions near the ventral tegmental area (VTA), where opiates inhibit local gamma-aminobutyric acid (GABA) neurons by acting on mu opioid receptors [2], leading to increased firing of dopamine neurons via disinhibition [3]–[4], and increased accumbal dopamine [5]–[6].

Opiate-induced dopamine elevations in the forebrain also depend, however, on cholinergic inputs from the pedunculopontine (PPT) and laterodorsal (LDT) tegmental nuclei that excite midbrain dopamine neurons [7]–[8]. In rats, PPT or LDT lesions reduce most of the striatal or accumbal dopamine increases following systemic morphine [9]–[10]. Additionally, VTA or SN infusions of the non-selective muscarinic receptor antagonist scopolamine similarly reduce most striatal or accumbal dopamine increases following systemic morphine [11]. PPT lesions also block morphine place preference [12] and heroin self-administration [13] in drug-naïve rats. The pathways through which the PPT mediates the acute rewarding effects of opiates and dopamine activation (i.e., via descending or ascending projections) are not clear, but cholinergic mechanisms are important. For example, intra-VTA infusion of the muscarinic antagonist atropine reduces morphine place preference in rats [14].

The M_5_ muscarinic acetylcholine receptor subtype, associated with dopamine neurons, is most important for cholinergic excitation of VTA and SN dopamine neurons [15]–[19]. In mice, for example, accumbal dopamine efflux following LDT electrical stimulation occurs in three phases. The strongest dopamine efflux in the long third phase is absent in M_5_ knockout mice [19].

M_5_ muscarinic excitation is also critical for opiate functions. M_5_ knockout mice show reduced morphine-induced accumbal dopamine efflux as measured by microdialysis, and reduced morphine conditioned place preference [20]. Also, morphine-induced locomotion is reduced by ∼40–50% in M_5_ knockout mice [21]. M_5_ muscarinic receptors in the VTA, therefore, contribute strongly to both the stimulant and rewarding effects of morphine.

The goal of the current studies was to better understand the role of the PPT and M_5_ receptors in opiate-induced dopamine activation. Increases in accumbal dopamine efflux following VTA infusions of morphine were absent in M_5_ knockout mice. Prolonged PPT-evoked striatal dopamine efflux, which has not been previously characterized in mice, was also reduced in M_5_ knockout mice. In both cases, M_5_-mediated increases in forebrain dopamine efflux followed a similar time course. Our results suggest that VTA opiates may indirectly activate dopamine neurons through a mechanism involving a PPT/LDT cholinergic relay.

## Results

### Experiment 1: Intra-VTA morphine-induced accumbal dopamine efflux in wildtype and M_5_ knockout mice

#### Histology

**Figure 1:**
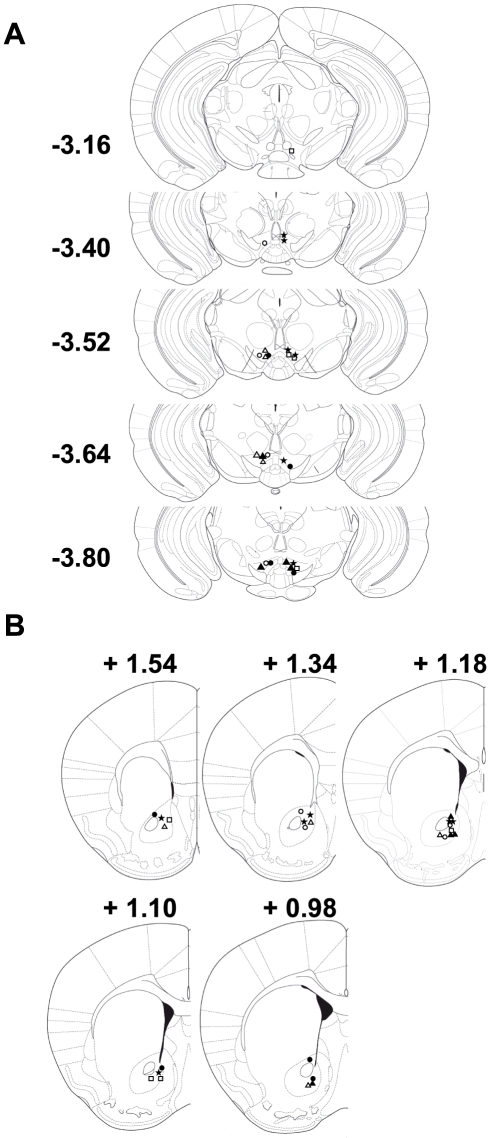
VTA injection sites (A) and nucleus accumbens recording sites (B) used in Experiment 1. In both (A) and (B), open circles show placements from M_5_ knockout mice (n = 4) treated with 50 ng intra-VTA morphine, closed circles show placements from M_5_ knockout mice treated with 0.3 µl intra-VTA saline (n = 4), open triangles show placements from wildtype mice (n = 4) treated with 50 ng intra-VTA morphine, closed triangles show placements from wildtype mice (n = 4) treated with 0.3 µl intra-VTA saline, stars show placements from wildtype mice (n = 6) pre-treated with 50 µg intra-VTA scopolamine followed by 50 ng intra-VTA morphine, and open squares show placements from wildtype mice (n = 4) pre-treated with 1 mg/kg (i.p.) naltrexone followed by 50 ng intra-VTA morphine. For clarity infusion sites are shown on both left and right side of the brain, but VTA infusions were always made on the left side of the brain. Numbers to the left of or above individual sections show distance in mm from bregma. Sections were adapted from the atlas of Paxinos and Franklin [38].

Figure 1A shows VTA injection sites identified from brains of individual wildtype and M_5_ knockout mice in Experiment 1. Injection sites in the various treatment conditions were confined within the boundaries of the VTA and were spread along its rostro-caudal extent. Recording sites in the nucleus accumbens (Fig. 1B) were confined to the core region between 0.98 and 1.54 mm anterior to bregma. Only mice with both VTA injection and accumbens recording sites were used for statistical analysis.

#### VTA morphine increases accumbal dopamine efflux in wildtype, but not in M_5_ knockout, mice

**Figure 2:**
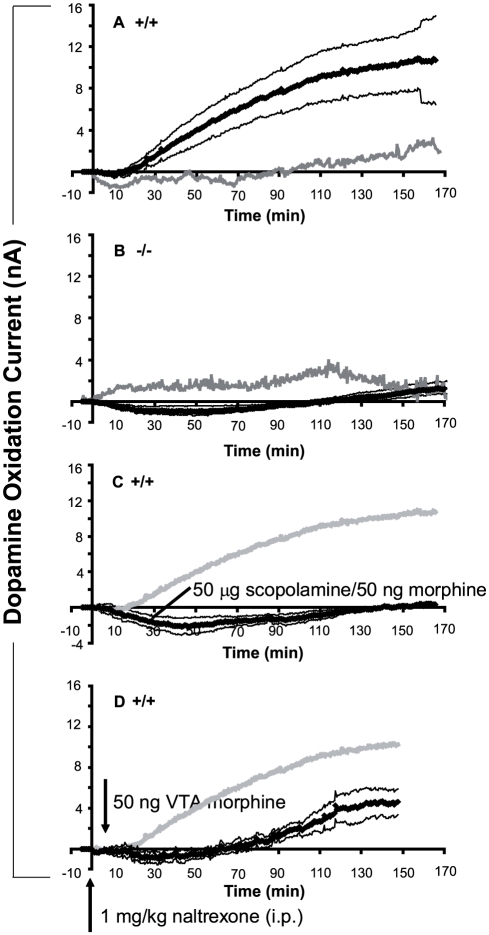
Changes in accumbal dopamine efflux produced by 50 ng intra-VTA morphine or saline in wildtype (+/+) or M_5_ knockout (−/−) mice. In all cases, thick lines represent mean dopamine oxidation current across mice, and thin lines represent ±SEM. (A) Mean change in accumbal dopamine efflux in wildtype mice following 50 ng intra-VTA morphine (black line, n = 4) or 0.3 µl intra-VTA saline (gray line, n = 4). (B) Mean change in accumbal dopamine efflux in M_5_ knockout mice following 50 ng intra-VTA morphine (black line, n = 4) or 0.3 µl intra-VTA saline (gray line, n = 4). (C) Effects of pre-treatment with 50 µg intra-VTA scopolamine on accumbal dopamine efflux induced by 50 ng intra-VTA morphine in wildtype mice (black line, n = 6). For comparison, data from (A) showing mean changes in accumbal dopamine efflux induced by 50 ng intra-VTA morphine without any pre-treatment in wildtype mice are reproduced (gray line, n = 4). (D) Effects of pre-treatment with naltrexone (1 mg/kg, i.p.) 5 min prior to 50 ng intra-VTA morphine in wildtype mice (black line, n = 4). For comparison, data from (A) showing mean changes in accumbal dopamine efflux induced by 50 ng intra-VTA morphine without any pre-treatment in wildtype mice are reproduced (gray line, n = 4).

Compared to saline, intra-VTA infusions of morphine (50 ng) in wildtype mice induced a delayed increase in accumbal dopamine efflux that began 16.5±7.9 min after infusion and steadily increased over the course of the following 2 hrs. Subsequently, dopamine efflux leveled off over a 50-min period, but never decreased back to baseline levels (Fig. 2A). By contrast, in M_5_ knockout mice the same dose of intra-VTA morphine induced a slight decrease in accumbal dopamine efflux relative to saline, starting at 5.9±2.0 min, that returned to pre-injection baseline levels by 92.0±9.8 min and subsequently showed a slight increase to above baseline over the final 30 min (Fig. 2B).

A between-within repeated-measures ANOVA with genotype as the between-subjects factor and time as the within-subjects factor revealed a significant interaction between genotype and time (F (344, 1376) = 11.59, p<0.0001), confirming that the temporal profile of accumbal dopamine efflux induced by intra-VTA morphine was different between wildtype and M_5_ knockout mice. Post-hoc analysis using Fischer's LSD test showed significantly lower accumbal dopamine efflux in M_5_ knockout mice at every 30-sec time point between 32 and 165 min (p's<0.05 to p's<0.001).

#### VTA scopolamine prevents increases in accumbal dopamine efflux induced by intra-VTA morphine in wildtype mice

In wildtype mice, intra-VTA pre-treatment with scopolamine (50 µg) prevented the increase in accumbal dopamine efflux typically observed following intra-VTA morphine (50 ng) (Fig. 2C). Most interestingly, the intra-VTA combination of scopolamine and morphine in wildtype mice resulted in a decrease in accumbal dopamine efflux, comparable to that observed following intra-VTA morphine alone in M_5_ knockout mice (Fig. 2B vs. 2C). A two-way between-within repeated-measures ANOVA with drug pre-treatment in wildtype mice (VTA scopolamine vs. no pre-treatment) as the between-subjects factor and time as the within-subjects factor revealed a significant interaction between pre-treatment and time, F (306, 2448) = 11.22, p<0.000001, indicating that the temporal profile of dopamine efflux varied as a function of VTA pre-treatment. Post-hoc analysis using Fischer's LSD test showed that scopolamine pre-treatment significantly reduced accumbal dopamine efflux at every 30-sec time point between 40 and 165 min (all p's<0.05).

#### Increased accumbal dopamine efflux induced by intra-VTA morphine is prevented by systemic naltrexone in wildtype mice

Figure 2D shows accumbal dopamine efflux induced by intra-VTA morphine (50 ng) in wildtype mice that were pre-treated with systemic naltrexone (1 mg/kg i.p.). VTA morphine infusion delivered 5 min after systemic naltrexone resulted in a decrease in accumbal dopamine efflux to below baseline levels that peaked at 33.7±4.7 min and returned to pre-injection baseline levels by 57.8±9.3 min. Over the course of the next 1.5 hrs, accumbal dopamine efflux gradually increased but always remained below levels observed in VTA morphine-infused wildtype mice that were not pre-treated with naltrexone. Thus, systemic naltrexone pre-treatment effectively blocked the steady morphine-induced increase observed in wildtype mice starting between 10–20 min, but was unable to block a small increase in dopamine efflux starting at approximately 1 hr. A two-way between-within repeated measures ANOVA with group (naltrexone pre-treatment vs. no pre-treatment) as the between-subjects factor and time as the within-subjects factor revealed a significant interaction between group and time (F (305, 1830) = 3.01, p<0.00001). This indicates that the temporal profile of accumbal dopamine efflux across the 2.5-hr recording period was different between mice pre-treated with naltrexone and mice that received no pre-treatment. Post-hoc analysis using Fischer's LSD test showed significantly reduced accumbal dopamine efflux in mice pre-treated with naltrexone at every 30-sec time point between 54 and 148 min (all p's<0.05).

### Experiment 2: PPT-evoked striatal dopamine efflux in wildtype and M_5_ knockout mice

#### Histology

**Figure 3:**
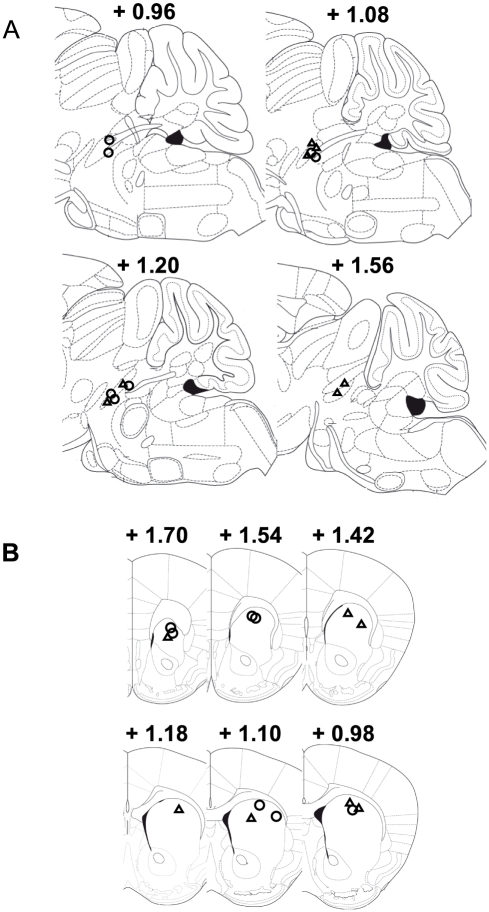
PPT stimulation sites (A) and striatal recording sites (B) used in Experiment 2. (A) Sagittal sections of the mouse brain showing tip placements of PPT stimulating electrodes in wildtype (open triangles, n = 7) and M_5_ knockout (open circles, n = 7) mice. Numbers above individual sections indicate lateral distance in mm from the midline. (B) Coronal sections of the mouse brain showing tip placements of recording electrodes in wildtype (open triangles, n = 7) and M_5_ knockout (open circles, n = 7) mice. Numbers above individual sections show distance in mm from bregma. Sections are adapted from the atlas of Paxinos and Franklin [38].

PPT stimulating electrodes in both wildtype and M_5_ knockout mice were distributed along the rostro-caudal extent of the PPT as shown on sagittal sections (Fig. 3A). Recording electrodes were confined within the boundaries of the striatum, between 0.98–1.70 mm anterior to bregma in both wildtype and M_5_ knockout mice (Fig. 3B). At the current used in these experiments, the stimulation was estimated to activate unmyelinated PPT cholinergic axons (conduction velocities of 0.4–1.2 m/s) within a distance of 0.75 mm of the electrode tip [22]–[23].

#### M_5_ knockout mice show reduced PPT-evoked striatal dopamine efflux

**Figure 4:**
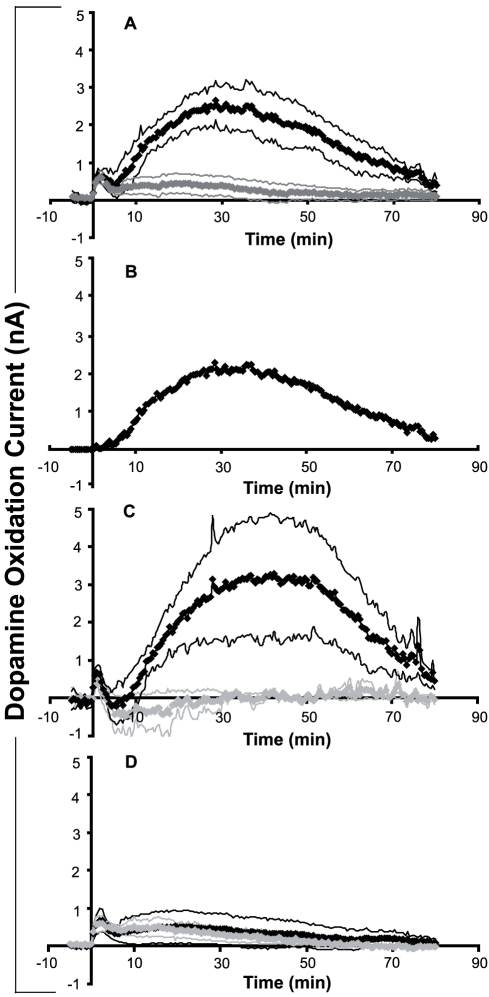
Striatal dopamine efflux following electrical stimulation of the PPT in wildtype and M_5_ knockout mice. In all cases thick lines represent mean oxidation current corresponding to striatal dopamine efflux across mice, and thin lines represent ± SEM. (A) PPT-evoked striatal dopamine efflux in wildtype (black trace, n = 7) and M_5_ knockout (gray trace, n = 7) mice. (B) M_5_ receptor contribution to PPT-evoked striatal dopamine efflux in mice. The graph shows the difference in dopamine efflux between wildtype (n = 7) and M_5_ knockout (n = 7) mice. (C) Effects of scopolamine (5 mg/kg, i.p.) pre-treatment on PPT-evoked striatal dopamine efflux in 4 of the wildtype mice shown in (A). The black trace shows PPT-evoked dopamine efflux, and the gray trace shows PPT-evoked dopamine efflux following systemic scopolamine. (D) Effects of scopolamine (5 mg/kg, i.p.) pre-treatment on PPT-evoked striatal dopamine efflux in 4 of the M_5_ knockout mice shown in (A). The black trace shows PPT-evoked dopamine efflux and the gray trace shows PPT-evoked dopamine efflux following systemic scopolamine.

Electrical stimulation of the PPT in wildtype mice induced two increases in striatal dopamine efflux separated by a trough near 5 min post-stimulation. The first increase began with the first measurement at 30 sec after stimulation and peaked at a mean of 1.25 min (Fig. 4A, black line and Table 1). This was followed by a more gradual decline in dopamine efflux (trough) that reached a minimum at 4.25 min. At ∼5–6 min after PPT stimulation, dopamine efflux began to increase again (second increase). This second increase peaked at 33 min and had a mean duration of 68.1±3.7 min, as measured by the time from the trough minimum to the time the signal returned to pre-stimulation baseline levels. This second increase was ∼3.4 times larger than the first increase in dopamine efflux and over 30 times longer in duration.

**Table 1:** Effects of PPT stimulation on dopamine oxidation current recorded from the striatum of wildtype (WT) and M_5_ knockout (KO) mice before and after systemic administration of scopolamine (5 mg/kg, i.p.).

		FIRST INCREASE		TROUGH		SECOND INCREASE	
Group (n)	Drug	Peak	Peak	Minimum	Minimum	Peak	Peak
		(nA)	Time (min)	(nA)	Time (min)	(nA)	Time (min)
**WT (7)**	**none**	0.86±0.2	1.21±0.2	0.17±0.3	4.14±0.3	3.24±0.5	34.17±4.2
**M5 KO (7)**	**none**	0.68±0.2	1.50±0.2	0.26±0.1	4.5±0.2	0.62±0.2*	27.33±7.9
**WT (4)**	**none**	0.74±0.3	1.25±0.4	0.11±0.6	4.25±0.4	3.86±1.4	36.62±5.8
**WT (4)**	**scopolamine**	0.63±0.3	1.12±0.1	−0.43±0.1†	4.75±0.2	0.45±0.1†	38.62±11
**M5 KO (4)**	**none**	0.69±0.3	1.75±0.4	0.36±0.2	4.62±0.2	0.62±0.4*	23.00±3.2
**M5 KO (4)**	**scopolamine**	0.61±0.2	1.62±0.6	0.44±0.1	4.37±0.6	0.61±0.2	15.13±3.5

*p<0.05 relative to wildtype control,

† p<0.05 relative to respective within-animal control.

PPT stimulation in M_5_ knockout mice also induced two increases in striatal dopamine efflux separated by a trough (Fig. 4A, gray line and Table 1). Neither the magnitude nor the time of the first increase or the subsequent trough in striatal dopamine efflux differed significantly between M_5_ knockout and wildtype mice (all p's>0.1). By comparison however, the second increase was markedly attenuated in M_5_ knockout mice, with peak dopamine efflux significantly lower compared to wildtype mice (t(14) = 3.38, p<0.01). The minimum of the trough was always above baseline in both wildtype and M_5_ knockout mice.

To calculate the overall M_5_ contribution to PPT-evoked striatal dopamine efflux, the average PPT-evoked change in basal dopamine oxidation current in M_5_ knockout mice was subtracted from the average change in wildtype mice. This shows that following electrical stimulation of the PPT, M_5_ receptors mediate a delayed increase in striatal dopamine efflux with the greatest rise occurring between 5 and 22 min, and a peak around 30 min (Fig. 4B). This delayed M_5_ contribution is consistent with previous studies measuring M_5_ time course in pilocarpine-induced salivation and in N-methyl-scopolamine binding studies *in vitro* [24]–[25].

#### Systemic scopolamine pre-treatment reduces striatal dopamine efflux in wildtype, but not in M_5_ knockout, mice

In wildtype mice, systemic administration of scopolamine 30 min prior to PPT stimulation did not alter the magnitude (t(3) = 1.00, p>0.3), or the time (t(3) = 0.39, p>0.7) of the first increase in striatal dopamine efflux. The trough was slightly enhanced, with the minimum now below baseline, resulting in a statistically significant difference relative to the pre-scopolamine baseline (t(3) = 3.94, p<0.05). Pre-treatment with scopolamine almost completely abolished the second increase in striatal dopamine efflux (t(3) = 7.53, p<0.01) in wildtype mice (Fig. 4C).

In M_5_ knockout mice (Fig. 4D), systemic administration of scopolamine 30 min prior to PPT stimulation did not affect the magnitude (p>0.5) or the time (p>0.5) of the first increase in striatal dopamine efflux. The trough was also not affected in M_5_ knockout mice, either in terms of its minimum (p>0.4) or the time of the minimum (p>0.2). Scopolamine had no effect on the already significantly reduced second increase in dopamine efflux in M_5_ knockout mice (p>0.9).

## Discussion

Increases in accumbal dopamine efflux following intra-VTA morphine were absent in M_5_ knockout mice and blocked by VTA scopolamine pre-treatment in wildtype mice. In a similar manner, the second increase in striatal dopamine efflux following electrical stimulation of the PPT was absent in M_5_ knockout mice and blocked by systemic scopolamine pre-treatment in wildtype mice. In both cases M_5_ receptors mediated a delayed onset, prolonged increase in accumbal and striatal dopamine efflux. Together, these data suggest that M_5_ receptor activation in the VTA/SN is critical for delayed, long-lasting excitation of dopamine neurons by either intra-VTA morphine or PPT electrical stimulation.

In wildtype mice, morphine infusion into the VTA induced a slow and long-lasting (>2 hrs) increase in accumbal dopamine efflux that began 10–20 min after infusion. This effect was entirely absent in M_5_ knockout mice. By contrast, nicotine or carbachol in rats has been shown to increase basal dopamine efflux in the accumbens within 1 min following VTA infusion [8], [26]–[27], indicating a 10–20 min delay in the activation of dopamine neurons by VTA morphine and consequent detectable increases in accumbal dopamine efflux.

Accumbal dopamine efflux rose steadily from 10–150 min following VTA morphine infusion and then leveled off between 2.5 to 3 hrs post-infusion. Previous chronoamperometric measurement of both i.v. morphine-induced accumbal and striatal dopamine efflux in urethane-anesthetized rats using similar recording electrodes showed a shorter-latency increase in dopamine efflux that peaked at ∼40 min and fully returned to pre-injection baseline levels between 2–3 hours [9]–[11]. Similarly, morphine-induced accumbal dopamine efflux measured by microdialysis in mice showed a peak between 60 and 80 min that gradually returned toward baseline between 2.5–3 hrs [20], [28].

The increased accumbal dopamine efflux induced by 50 ng intra-VTA morphine observed in urethane-anesthetized mice is thought to have functional relevance, as mice self-administer a 50 ng dose of morphine into the VTA, and this self-administration has been shown to be reduced by pre-treatment with 4 mg/kg (i.p.) of the non-selective opioid receptor antagonist naloxone [29]. In a similar manner, we observed a marked reduction in the increase in accumbal dopamine efflux following pre-treatment with 1 mg/kg (i.p.) naltrexone. As morphine was infused directly into the VTA, it is likely that systemic naltrexone inhibited increases in dopamine efflux via blockade of VTA opioid receptors. Although naltrexone pre-treatment completely blocked morphine-induced accumbal dopamine efflux for the first 60 min, dopamine efflux recovered to above baseline levels over the course of the next 90 min (see Fig. 2D). Interestingly, pre-treatment with 1 mg/kg naltrexone in B6 wildtype mice has been shown to completely block morphine-induced locomotion (30 mg/kg, i.p.) for the first 90 min, but over the final 30 min of testing locomotor levels increase [21]. Thus, opioid receptors are likely responsible, in a similar way, for both the effects of morphine on locomotion in freely-behaving mice and on accumbal dopamine efflux induced by intra-VTA morphine.

Our previous chronoamperometry study showed that electrical stimulation of the LDT in wildtype mice evoked an initial brief increase in accumbal dopamine efflux, followed by a brief decrease to below baseline levels, and thereafter by a prolonged second increase in accumbal dopamine efflux [19]. The present study showed that electrical stimulation of the PPT in the same wildtype mice also evoked two comparable increases in striatal dopamine efflux. The first increase following PPT stimulation was not affected in M_5_ knockout mice. The second, prolonged increase began 5–6 min following stimulation, and in marked contrast to wildtype mice, the second increase in striatal dopamine efflux was significantly reduced in M_5_ knockout mice. In addition, scopolamine pre-treatment in wildtype mice prevented the second increase in striatal dopamine efflux. Together, these data suggest that M_5_ receptors in the SN [15]–[16] play a critical role in mediating sustained excitatory cholinergic input to the nigrostriatal dopamine system.

The trough in striatal dopamine efflux following PPT stimulation was reduced compared to LDT stimulation. Following LDT stimulation in rats, the trough in accumbal dopamine has been shown to be mediated by M_2_-like autoreceptors at the level of the LDT [17]. The relatively smaller trough observed here in the striatum suggests that these autoreceptors may not play a significant role in regulating PPT cholinergic inputs to the SN under the current conditions. In addition, the second increase in PPT-evoked striatal dopamine efflux was much larger (∼3.4-fold) and faster (∼5 min) relative to the first increase, compared to LDT-evoked accumbal dopamine efflux (∼2-fold and ∼8 min) described previously [19]. This suggests that the trough in PPT-evoked striatal dopamine efflux in 129 wild-type mice is partly masked by a larger M_5_-mediated second increase in prolonged striatal dopamine efflux. Inspection of Figure 4B supports this as the M_5_ contribution to increased striatal dopamine efflux following PPT stimulation began during the trough (i.e. between 5 and 22 min), thus likely masking the inhibitory effect of the trough.

The temporal changes in dopamine efflux following PPT or LDT stimulation provide several similarities to the effects of intra-VTA morphine on accumbal dopamine efflux observed in Experiment 1. Electrical stimulation of the PPT (Experiment 2) increased striatal dopamine efflux with a relatively rapid onset (<30 sec) followed by an M_5_ receptor-dependent increase in dopamine efflux occurring ∼5 min after stimulation (see Fig. 4B). By comparison, intra-VTA morphine also increased accumbal dopamine efflux in a similar delayed manner, and only in wildtype mice (Fig. 2A). If opiates in the VTA were affecting mesolimbic dopamine neurons exclusively through disinhibition of local GABA neurons [2], then elevations in basal extracellular dopamine concentrations would be expected to occur faster than 10 min. Instead, the present data suggest that the 10-min onset delay was due to opiate-induced disinhibition of PPT and LDT cholinergic neurons, which, in turn, act more slowly on midbrain dopamine neurons via M_5_ muscarinic receptors (see Fig. 4B). Opiates can inhibit GABA neurons in the VTA [2] and descending GABAergic projections to the PPT are important for opiate reward in drug-naïve animals [30]. SN pars reticulata GABA neurons have been shown to directly inhibit cholinergic neurons in the pons [22] and whole-cell patch clamp recordings have shown that cholinergic neurons in the LDT are inhibited by GABA application [31]. Conceivably, inhibition of GABAergic projections to the PPT and LDT by VTA opiates may, in turn, lead to a disinhibition of ascending PPT/LDT cholinergic inputs to the SN/VTA thereby enhancing dopamine neurotransmission via activation of M_5_ receptors. The indirect activation of midbrain dopamine neurons through a mesopontine cholinergic relay offers an explanation for the delayed onset action of intra-VTA morphine. This mechanism also explains the inability of intra-VTA morphine to enhance basal accumbal dopamine efflux in M_5_ knockout mice, as well as the ability of intra-VTA scopolamine to effectively block this increase in wildtype mice.

Our morphine infusions sites tended to be in the caudal half of the VTA (Fig. 1A), so it is possible that the 0.3 µl infusions could have spread to affect GABA neurons in the neighboring rostromedial tegmentum (RMTg), where high levels of mu opioid receptors are found [32]. Similar to the VTA, RMTg neurons can be inhibited by morphine [33], and RMTg neurons project to, among other places, the VTA and SNc, as well as the PPT [32].

In conclusion, the present data show that prolonged increases in accumbal dopamine efflux induced by intra-VTA morphine, and prolonged increases in striatal dopamine efflux induced by PPT electrical stimulation both critically depend on M_5_ muscarinic receptors in the midbrain. Consistent with previous studies [19]–[20], [34], the present data further emphasize the importance of M_5_ receptors in modulating cholinergic excitation of both nigrostriatal and mesolimbic dopaminergic transmission. Previous studies have shown that the PPT/LDT [9]–[10] and muscarinic receptors in the SN/VTA [11] are critical for morphine-induced dopamine activation. Here we show a complete loss of increased dopamine efflux induced by intra-VTA morphine in M_5_ knockout mice. Taken together, these data suggest an important role for increased cholinergic input to the VTA in mediating dopamine activation induced by morphine and specifically suggest that M_5_ receptors are a critical target through which opiates in the VTA activate mesencephalic dopaminergic systems.

## Materials and Methods

### Mice

M_5_ knockout mice were homozygous mutants for the M_5_ muscarinic acetylcholine receptor gene. Mice were created using recombinant DNA methods on a mixed 129 SvJ×CD1 background as described by Takeuchi et al. [25]. Homozygous and wildtype mice were used to maintain a colony based on homozygous breeding. In all experiments litters of male homozygous M_5_ knockout and wildtype mice were age-matched (4–8 months at the time of testing). Eighteen male wildtype and 8 male M_5_ knockout mice were used in Experiment 1. Seven wildtype and 7 M_5_ knockout mice were used in Experiment 2. These studies were approved by the University of Toronto Animal Care Committee under protocol # 20007696.

### Stearate-modified carbon-paste electrodes

Stearate-modified carbon paste electrodes were constructed according to the methods of Blaha and Jung [35]. Briefly, 75 mg of stearate (99.9% purity, Sigma-Aldrich, St. Louis, MO) were dissolved in 1 ml silicone oil (Sigma-Aldrich, St. Louis, MO) heated to 40°C. To this solution 1 g of graphite powder (particle size <20 µm, Sigma, St. Louis, MO) was added and thoroughly mixed until reaching a paste-like consistency. Electrodes were constructed by packing carbon paste into a 0.5–1 mm well, created by extruding the Teflon coating off a 0.008″ o.d. Teflon-coated stainless-steel wire (Medwire, Mount Vernon, NY) beyond the tip of a ∼10 cm length of wire. The carbon paste was packed into the well tightly by dropping the electrode tip on a glass plate several times. The surface of the electrode tip was investigated under a microscope to ensure that there were no cracks or grooves in the surface of the carbon paste.

### Experiment 1: Morphine-induced accumbal dopamine efflux in wildtype and M_5_ knockout mice

#### Surgery

Mice were anesthetized with urethane (1.5 g/kg, i.p.). Each mouse was mounted in a stereotaxic frame (David Kopf Instruments, Tujunga, CA or MyNeuroLab, St. Louis, MO) using rat earbars (Stoelting, Wood Dale, IL) and a mouse head-holder (Stoelting, Wood Dale, IL). Temperature was maintained at 37±0.5°C with a temperature-regulated heating pad (TC-1000; CWE Inc., New York, NY). A single 26 gauge guide cannula (Plastics One, Roanoke, VA) was implanted 1 mm dorsal to the left VTA of each mouse (A/P +0.9 mm from lambda, M/L 0.4 mm D/V −4.4 mm from dura). For VTA infusions a 33 gauge cannula was inserted 1 mm past the tip of the guide cannula, and was connected to a Hamilton microsyringe via Tygon tubing. A single stearate-modified carbon paste recording electrode was implanted into the left nucleus accumbens of each mouse (A/P +1.4 mm from bregma, M/L 0.8 mm, D/V −3.6 mm from dura). A combination Ag/AgCl reference and stainless-steel auxiliary electrode was placed into contact with the contralateral parietal cortex.

#### Electrochemical recordings and VTA microinfusions

After *in vivo* implantation of all electrodes and cannulae, the working characteristics of the recording electrode were evaluated by recording several voltammetric sweeps in the striatum (triangular wave potentials applied from −0.15 to 0.45 V vs. Ag/AgCl; ramp rate 10–20 mV/sec) using an electrometer (EChempro; GMA Technologies, Vancouver, Canada). After confirming the presence of a dopamine peak in the voltammogram, repetitive chronoamperometric measurements of oxidation current were made by applying a potential pulse from −0.15 V to +0.25 V to the recording electrode for 1 sec at 30-sec intervals and monitoring the current for the final 50 ms of each 1-sec pulse [36]. Following at least 30 min of baseline recordings, VTA infusions of morphine (50 ng, 0.3 µl volume) were made and changes in accumbal dopamine oxidation current, corresponding to dopamine efflux, were monitored for 2–3 hrs thereafter. Changes in dopamine efflux following 50 ng intra-VTA morphine were tested in 4 wildtype and 4 M_5_ knockout mice. In an additional 6 wildtype mice, changes in accumbal dopamine efflux in response to 50 ng intra-VTA morphine were measured following VTA pre-treatment with 50 µg scopolamine 10 min prior to morphine. Changes in accumbal dopamine efflux following 50 ng intra-VTA morphine were also measured in an additional 4 wildtype mice following systemic pre-treatment with naltrexone hydrochloride (1 mg/kg, i.p.) 5 min prior to morphine. Finally, changes in accumbal dopamine oxidation current in response to 0.3 µl VTA saline were measured in 4 wildtype and 4 M_5_ knockout mice.

#### Data Analysis

Pre-stimulation baseline chronoamperometric currents were normalized to zero current values, with induced changes in oxidation current, corresponding to changes in dopamine efflux, presented as absolute changes in current (increases as positive and decreases as negative). Changes in dopamine efflux following intra-VTA morphine in wildtype and M_5_ knockout mice, or following combinations of intra-VTA morphine and intra-VTA scopolamine or systemic naltrexone in wildtype mice were analyzed using mixed-model ANOVA. Significant interactions were further analyzed using Fisher's LSD test.

### Experiment 2: PPT-evoked striatal dopamine efflux in wildtype and M_5_ knockout mice

#### Surgery

Surgical procedures and chronoamperometric recordings followed those described for Experiment A single concentric, bipolar stimulating electrode (SNE-100; Rhodes Medical Co., Woodland Hills, CA) was implanted into the left PPT of each mouse (A/P −0.5 mm from lambda, M/L 1.2 mm, D/V −2.9 mm from dura) according to the atlas of Paxinos and Franklin (2004), and a single stearate-modified carbon paste electrode was implanted into the left striatum (A/P +1.4 mm from bregma, M/L 1.5 mm, D/V −2.3 mm from dura). A combination Ag/AgCl reference and stainless-steel auxiliary electrode was placed into contact with the contralateral parietal cortex.

#### PPT electrical stimulation

Following at least 30 min of baseline recordings, a series of cathodal monophasic current pulses (400 µA, 0.5 ms duration) were delivered to the concentric, bipolar stimulating electrode implanted in the PPT via a programmable pulse generator (Master-8; A.M.P.I., Jerusalem, Israel) in combination with a constant current stimulus isolation unit (Iso-Flex; A.M.P.I., Jerusalem, Israel). Each stimulation of the PPT consisted of a 1 sec, 35 Hz train of 400 µA pulses (1 sec inter-train interval) applied over a 60 sec period (1050 pulses in total). These parameters were designed to mimic spontaneous firing patterns of PPT neurons in awake, naturally aroused animals [37]. In urethane-anesthetized rats these stimulation parameters (at 800 µA) have been shown to evoke a tri-phasic pattern of striatal dopamine efflux [18].

#### Systemic drug administration

Subsequent to monitoring the effects of PPT stimulation on striatal dopamine efflux, a sub-set of mice (4 wildtypes and 4 M_5_ knockouts) was injected with the non-selective muscarinic receptor antagonist scopolamine hydrobromide (5 mg/kg, i.p.). Thirty min later, the PPT was again stimulated and changes in striatal dopamine oxidation currents were monitored for an additional 80 min.

#### Data analysis

Pre-stimulation baseline chronoamperometric currents were normalized to zero current values, with stimulated change in oxidation current, corresponding to changes in dopamine efflux, presented as absolute changes in current (increases as positive and decreases as negative). The maximal and minimal PPT-evoked changes in dopamine efflux associated with the two increases and the decrease, respectively, were obtained for individual wildtype and M_5_ knockout mice. In the case of the 8 mice that were pre-treated with scopolamine, maximal and minimal PPT-evoked changes in dopamine efflux after scopolamine were also obtained and compared to pre-scopolamine PPT-evoked values. Mean peak efflux (in nanoamperes, nA) and peak times (in min) were compared between wildtype and M_5_ knockout mice (two-tailed unpaired t-test) and before and after scopolamine in all mice (two-tailed paired t-test). The peak time of the second increase in dopamine efflux, which was strongly attenuated in M_5_ knockout mice, was not statistically analyzed.

To calculate the overall M_5_ contribution to PPT-evoked striatal dopamine efflux, the average PPT-evoked change in basal dopamine oxidation current in M_5_ knockout mice was subtracted from the average change in wildtype mice at each 30-sec time point. The calculated differences were then plotted as a function of time following PPT stimulation.

### Histology

On completion of all experiments mice were killed with a 0.25 ml intracardial injection of urethane. Their brains were removed and put into a 10% formalin solution. All brains were then sectioned on a Vibratome, examined under a light microscope, and compared to atlas sections (Paxinos and Franklin, 2004).

On completion Experiment 2, a PPT lesion was made by passing direct current (100 µA for 5 sec) through the concentric bipolar stimulating electrode. For these brains 0.1% potassium ferricyanide was included in the formalin solution. The iron deposited at the stimulating electrode tip by the electrolytic lesion reacted with the potassium ferricyanide to form a Prussian blue spot, clearly marking the PPT stimulation site.

### Drugs

Morphine sulfate pentahydrate, scopolamine hydrobromide, naltrexone hydrochloride, and urethane were purchased from Sigma Aldrich (St. Louis, MO) and dissolved in sterile 0.9% saline. Drugs were infused into the VTA or systemically injected as their salt weight. Intra-VTA infusions were made at a volume of 0.3 µl, and systemic naltrexone and scopolamine injections at a volume of 10 ml/kg.
